# Authors' Response to Peer Reviews of “Cognitive Factors Associated With Public Acceptance of COVID-19 Nonpharmaceutical Prevention Measures: Cross-sectional Study”

**DOI:** 10.2196/37241

**Published:** 2022-05-13

**Authors:** Aymery Constant, Donaldson Conserve, Karine Gallopel-Morvan, Jocelyn Raude

**Affiliations:** 1 Ecole des Hautes Etudes en Santé Publique Rennes France; 2 Department of Prevention and Community Health Milken Institute School of Public Health George Washington University Washington, DC United States

**Keywords:** Extended Parallel Process Model, COVID-19, lockdown, public acceptance, nonpharmaceutical measures, Likert scale, France

*This is the authors’ response to peer-review reports for “Cognitive Factors Associated With Public Acceptance of COVID-19 Nonpharmaceutical Prevention Measures: Cross-sectional Study.”*

## Round 1 Review

We are grateful to the reviewers [1,2] for the truly helpful comments they made when revising the previous manuscript. We did our best to be receptive when revising our paper [3]. Please find below a detailed point by point response. Please note that all changes are marked in bold in the revised manuscript.

### Specific Comments

1. Your title needs to follow the guidelines of the journal to which you are submitting.

Response: We have revised the title to reflect the guidelines of the journal.

2. The “Background” and “Methods” subsections of your Abstract need to be improved.

Response: As recommended, the Background and Methods subsections of the Abstract have now been improved.

3. The specific objectives of the paper need to stand out as a subsection.

Response: We have added the specific objectives of the paper as a subsection.

4. Major subsections are missing in your introduction, methods, and the results.

Response: We have added the subsections in the different sections of the manuscript.

5. Some subsections in the Methods section warrant improvement.

Response: We have revised the subsections of the Methods section based on additional feedback from the Major comments section.

6. The structure of the Discussion section needs to align with the guidelines.

Response: We appreciate the suggestion and have aligned the Discussion section with the guidelines.

7. The in-text citations and references must comply with the journal’s guidelines.

Response: The citations and references now comply with the journal’s guidelines.

8. Tables and figures in the appendix need to be moved to the body of the text.

Response: We appreciate the suggestions and have revised the title and abstract in response to the specific comments.

#### Major Comments

1. Format your title to include the country and study design. Kindly refer to the guidelines for titles [4]. For instance, “Acceptance of COVID-19 preventive measures as a trade-off between health and social outcomes in France: Cross-sectional Study”. By the way, I have not seen anywhere in the body of your paper where health and social outcomes mentioned in your title have been articulated.

Response: We have changed the title to reflect the recommendation and remove the health and social outcomes that are not mentioned in the paper.

2. The beginning of your background in the Abstract (“A better understanding of the factors underlying their acceptance may contribute greatly to the design of more effective public health programs during the current and future pandemics”) does not make it clear to the reader to whom you are alluding. Kindly rephrase.

Response: As recommended, we have revised the sentence to clarify the point we aimed to make.

3. Your objectives need to be improved. I guess along the lines of (1) measure the public’s acceptance of COVID-19 preventive measures and (2) assess the association of the public’s acceptance of these measures and their perception of COVID-19.

Response: We are grateful for the suggestion on how to improve the objectives. We have revised the objectives accordingly.

4. In the “Methods” subsection of your Abstract, kindly add a summary of how data for each objective was analyzed and the statistical package that was used to perform the analysis. Please note that your Abstract (currently <250 words) can go up to a maximum of 450 words. Response: We have revised the Methods subsection accordingly to reflect how the data were collected and analyzed for each objective as shown below.

5. It would be good to include the following items under Introduction after the background: (1) study rationale, to justify your study and to present the Extended Parallel Process Model, and (2) specific objectives, to clearly outline your study objectives.

Response: We agree with the suggestion and have added the subsections under Introduction.

6. Kindly start your Methods section with a subsection “Study Design” and specify your study design.

Response: Study Design has been added as the first subsection in the Methods section.

7. The statement under Participants and Procedures—that is, “The objective of the research was to assess the emotional, cognitive, and behavioral responses of the French people to the COVID-19 epidemic during the full lockdown (wave 1) and thereafter (wave 2)”—should not be there. You might want to move this to the study aim or specific objectives.

Response: We agree and have made the revision.

8. The second to last statement under Participants and Procedures (“For this study, we analyzed data from a 2-week survey administered 6-8 weeks after the first lockdown between June 25 and July 5, 2020”) does not fit quite well under this subsection. I suggest you rephrase as “This was a 2-week survey administered 6-8 weeks after the first lockdown of June 25 through July 5, 2020” and incorporate it into your Study Design subsection.

Response: We have added the suggestion to the Study Design subsection.

9. The last sentence under Participants and Procedures needs to be moved to a section entitled “Ethical Considerations” to be created at the end of the Methods section (just before the Results section).

Response: We agree and have moved the sentence to an “Ethical Considerations” subsection.

10. Kindly start your Results section with the subsection “Participant Characteristics” to give a summary of participant characteristics. Kindly move your Table 1 in the appendix to accompany your participant characteristics.

Response: As suggested, the Results section now starts with Participant Characteristics.

11. You need to move Tables 2-4 in the appendix to where they are first mentioned in the Results section for easy comprehension. It becomes easy to refer to the tables while reading. In addition, bear in mind that you are allowed to include up to a total of 5 tables in the body of your text.

Response: We have moved Tables 2 to 4 where they are first mentioned in the Results section.

12. Move Figure 1 to where it is first mentioned in your Results section.

Response: We have uploaded Figure 1 as a spare file, in accordance with journal guideline. It is indicated in the Results section where it should be inserted.

13. Kindly organize your Discussion into (1) Principal Results, (2) Comparison With Prior Studies, (3) Study Limitations, and (4) Conclusion.

Response: The Discussion is now organized as suggested.

14. The in-text citations and references must be in line with the AMA citation style, in accordance with the journal guidelines [5]. Kindly refer to the references accompanying this report.

Response: The in-text citations and references are now in accordance with the journal guidelines.

#### Minor Comments

15. Based on your title, I guess your study aimed to evaluate the acceptance of COVID-19 nonpharmaceutical measures. I suggest you add to your background (both in the Abstract and the Introduction) a study aim similar to the above and use the last sentence of your background in the Abstract to create a separate “Objectives” subsection before the Abstract’s “Methods” subsection.

Response: We have revised the background in the Abstract and Introduction accordingly.

16. I suggest you rephrase sentence #2 in the methods subsection of your Abstract as “For objective 1, participants were asked the extent to which they supported 8 COVID-19 preventive measures using a 4-point Likert scale”, and start the following sentence with “For objective 2, COVID-19 perceptions…”

Response: We have added the suggestions in the Methods subsection of the Abstract.

17. In the results subsection of the Abstract, could you please include figures for positive and negative associations and highlight if these were statistically significant or not?

Response: The results subsection of the Abstract now includes the figures for positive and negative associations and whether they were statistically significant or not.

18. Kindly include “Likert scale”, “France” and “Nonpharmaceutical measures” in your keywords.

Response: We have added the suggestions to the keywords.

19. Under Measurements, kindly substantiate your use of the Likert scale with suitable references. You might want to use this link [6].

Response: The above reference is now inserted in the manuscript.

20. For your beginning statement under Data Analysis, I suggest you use “frequencies (N)” instead of “numbers (N)”.

Response: The term “frequencies” is now used in the Data Analysis section.

21. I like the flow and harmony between Participants and Procedure, Measurements, and Data Analysis. You did well to have organized these by objective. In your Data Analysis, could you please highlight how you assessed the model fit (goodness of fit) of your multivariate model?

Response: The goodness of fit for each multivariate model (value/df for the deviance) is now indicated (revised Tables 3 and 4).

22. I suggest you organize your Results section, which already is in good shape, by study objective after “Participant Characteristics” so that it flows well in the measurements and data analysis subsections.

Response: We appreciate the suggestion and have organized the Results section by study objective.

23. Relating your study results to the title, readers might expect to see where you articulated the trade-off between health and social outcomes. This is not the case. It might be worthwhile to rephrase your title.

Response: We have revised the title.

24. Kindly format your tables and figures following the journal guidelines.

Response: The tables are now edited according to journal guidelines.

25. I suggest you start your Conclusion by highlighting the study objectives.

Response: As suggested, we have revised the Conclusion and highlighted the study objectives.

26. It is important to include citations from the journal to which you are submitting or its sister journals.

Response: We have inserted 8 additional citations from JMIRx Med or sister journals in the manuscript.

## Round 2 Review

Again, we are grateful to the reviewer for the helpful comments and suggestions and believe that responding to them has resulted in an improved manuscript. Questions and concerns noted by the reviewer are addressed below.

### Major Comments

1. The phrase “The aim of this study was to evaluate the acceptance of COVID-19 nonpharmaceutical prevention measures in France”, in the Objectives subsection should be moved to be the last sentence of the Background subsection in your Abstract.

Response: The sentence was moved to be the last sentence of the Background subsection in the Abstract.

2. Under Rationale, I think you should start the second sentence as “This study was based on the Extended Parallel Process Model.”

Response: The sentence was inserted in the text as recommended by the reviewer (page 4).

3. The last sentence of your Rationale is not suitable for this section, so I suggest removing it.

Response: This last sentence of the rationale was removed.

4. The starting sentence of your Specific Objectives should be part of your Rationale instead, so you may want to move that from there.

Response: The starting sentence of the Specific Objectives (“As nonpharmaceutical interventions play a considerable role…) was moved to the Rationale (page 4).

5. All weblinks in the body of your text should be cited as references. The journal to which this manuscript is submitted does not allow the use of weblinks in the body of the text.

Response: All weblinks were removed from the text.

6. The phrases “EPPM factors were estimated using an unweighted least-square factorial analysis, followed by a Promax rotation, and 5 factors were extracted accordingly” and “The raw scale scores were transformed to a 0-100 scale. Higher scores in the respective scales are indicative of greater perceived efficacy, lack of fear control, severity, susceptibility, or avoidance” should be moved to Data Analysis.

Response: These sentences were moved to the methods section (page 8).

7. Tables 1, 3, and 4 still need to be updated to comply with the journal guidelines. You will notice in this link [7] that item categories like “Age in years” and “Professional status” should be in their own row while the items under each category start on the next row.

Response: Tables 1, 3, and 4 now comply with the guidelines. Thank you for the guidance.

8. As part of the participant characteristics, kindly include the mean age of participants and if the mean age difference between men and women was statistically significant.

Response: It is now stated in the Results section that “The mean age (SD) was 46.9 (SD 15.9) years, and was similar between men (mean 46.4, SD 16.3 years) and women (mean 47.4, SD 15.5 years; *P*=.18)” (page 9). It is also stated in the Methods section that numerical data were compared with a 1-way ANOVA (page 8).

9. Regarding your statement “The raw scale scores were transformed to a 0-100 scale”, there is a serious debate about calculating Likert scale scores from responses. Kindly be clear on how you converted the responses to scores.

Response: It is now stated in the method section that “EPPM raw scale scores were transformed to a 0-100 scale: ([raw score − lowest possible raw score]/possible raw score range) × 100” (page 8).

10. Kindly include your Figure 1 in the body of the text. All figures uploaded online must also be included in the body of the text, as per the guidelines.

Response: Figure 1 is now included in the body of the text (page 12).

11. Kindly move the first sentence of your Principal Results (“The aim of this study was to evaluate the acceptance of COVID-19 nonpharmaceutical measures and, more specifically, to measure the public’s acceptance of these measures and their association with COVID-19 perceptions”) to be the starting sentence of your Conclusion.

Response: The sentence was moved to be the starting sentence of the conclusion (page 17).

12. Kindly ensure that all percentages reported in the body of your text (apart from those from other studies) are expressed in absolute values in parentheses; for instance, 20% (5/25).

Response: All percentages (except for averages) are now expressed in absolute values (Results section, pages 9-10).

13. Evidence suggests that there are also issues around sex and gender reporting [8-10]. Since sex is biological, it will be good to make clear in your methods that the sex definition was based on self-reported sex [9].

Response: It is now mentioned in the Methods section that respondents had to report their gender (self-reported sex, page 7). Estimates for “female gender” are now reported in Tables 1, 3, and 4 for clarity.
